# Supplementary material for: Patterns of Intron Gain and Loss in Fungi
Source: PLoS Biol. 2004 Nov 30;2(12):e422. doi: 10.1371/journal.pbio.0020422 (PMC532390; doi:10.1371/journal.pbio.0020422)
Supplement: Table S1 — Also available at http://genes.mit.edu/NielsenEtAl/. (4.3 MB ZIP). [file pbio.0020422.st001.zip › NielsenEtAl/html/319.html]

AN3055.1.NCU09707.1.MG03855.1.FG06315.1


```
 CLUSTAL W (1.82) Multiple Sequence Alignments - Introns Inserted


Sequence 1: NCU09707.1	237 aa
Sequence 2: MG03855.1	236 aa
Sequence 3: FG06315.1	231 aa
Sequence 4: AN3055.1	250 aa
Alignment Length: 252 aa
Number Identitical Residues: 87 aa
Alignment Score (without introns) 5085


MG03855.1 	MPRLPNSEDPPERPDFITNIINGLERYNPEAVGTLEQYLTTQCEERFCDCNANRTLLKL2
NCU09707.1	----MNGEDPQERPDFIRAIINGLERYNPEAAGTLEAYLTQQCEEKFCDCNANRALLKL2
FG06315.1 	----MNGEDPPERPQEISSIINGLERYNPEAVGALETYLQDQCEQKFTDCNANRTLLKL2
AN3055.1  	--MGVAIDKCETRPANIEAILSGLDRYNPETTTVFQDYVVQQCEDRTFDCYANLALLKL2
          	       :.   **  *  *:.**:*****:. .:: *:  ***::  ** ** :**** 

MG03855.1 	YQLNPDRIKDEVITNILVKAMTLFPSPQFSQALHLL-------SPS----ALSQQSELSE
NCU09707.1	YQLNPDRIKDEVITNILVKAMTQFPSPQFDLALHLL-------SPSQSNPGPNSSSELTE
FG06315.1 	YQLNPDRIKDEVITNVLVKTMTQFPSAQFSLALHLI-------NPS-----SVVTGDLGE
AN3055.1  	YQFNPHLLQPETVTNILVKALTVFPSPAFSLCLALLPAYTQPFPSSEAEATAAQMSDFVE
          	**:**. :: *.:**:***::* ***. *. .* *:.: :..  .*.:..     .:: *

MG03855.1 	AVSKLRALNNQLEGAQYARFWATIESDDLYADLTTDIQGFEEMVRLRIAVLVSQAFREVQ
NCU09707.1	AVSKLRALNAQLEGAEYARFWATLDSDDLYADLTTDIAGFEDMIRVRIAQLVGQSYREIQ
FG06315.1 	AITKLRSLNGQLEGAQYSRFWADLD-DDMVADLIADIPDFEEVVRHRIALLVSQAFREIQ
AN3055.1  	SVQKLARLSSLLESAQYAQFWSTLNSDDLYADLVADVAGFEELVRIRIAVEVGKAFREVN
          	:: **  *.  **.*:*::**: :::**: *** :*: .**:::* ***  *.:::**::

MG03855.1 	LSLMEQWLGLDEAPLKT-FITEACGFKIEGD----IVQIPKNPDNEAKKAEIREDVNVEM
NCU09707.1	FPVLESWLGLNNSEATTQFITETCGWKVEGD----VVQIPKNADNEARKAEIREDVNVDM
FG06315.1 	IGHLESWLGLNEDATKK-FVTEVCGWTVDAEG---NVKVPSNPDNEAKKAEIREDVNVEQ
AN3055.1  	AEVLEQWLDVRNSEALEKFVTEVCSWEVDKSGSATVVKVPTNKENEARSEVKSERVGVDM
          	   :*.**.: :     .*:**.*.: :: ..::: *::*.* :***:.    * *.*: 

MG03855.1 	FSRVIRRAWEEVA
NCU09707.1	FARVIKRSWEESA
FG06315.1 	FSRVLRRSWEDTV
AN3055.1  	FGRVIRRGFEQAA
          	*.**::*.:*: .
```
